# Dual Drug Release Electrospun Core-Shell Nanofibers with Tunable Dose in the Second Phase

**DOI:** 10.3390/ijms15010774

**Published:** 2014-01-08

**Authors:** Wei Qian, Deng-Guang Yu, Ying Li, Yao-Zu Liao, Xia Wang, Lu Wang

**Affiliations:** 1 School of Materials Science & Engineering, University of Shanghai for Science and Technology, Shanghai 200093, China; E-Mails: qianwei@usst.edu.cn (W.Q.); liying516@126.com (Y.L.); ydg017@usst.edu.cn (L.W.); 2 School of Chemistry, University of Bristol, Bristol BS8 1TS, UK; E-Mail: yaozu.liao@bristol.ac.uk

**Keywords:** modified coaxial electrospinning, core-shell nanofibers, dual drug release, Teflon-coated spinneret, tunable dose

## Abstract

This study reports a new type of drug-loaded core-shell nanofibers capable of providing dual controlled release with tunable dose in the second phase. The core-shell nanofibers were fabricated through a modified coaxial electrospinning using a Teflon-coated concentric spinneret. Poly(vinyl pyrrolidone) and ethyl cellulose were used as the shell and core polymer matrices respectively, and the content of active ingredient acetaminophen (APAP) in the core was programmed. The Teflon-coated concentric spinneret may facilitate the efficacious and stable preparation of core-shell nanofibers through the modified coaxial electrospinning, where the core fluids were electrospinnable and the shell fluid had no electrospinnability. The resultant nanofibers had linear morphologies and clear core-shell structures, as observed by the scanning and transmission electron microscopic images. APAP was amorphously distributed in the shell and core polymer matrices due to the favorite second-order interactions, as indicated by the X-ray diffraction and FTIR spectroscopic tests. The results from the *in vitro* dissolution tests demonstrated that the core-shell nanofibers were able to furnish the desired dual drug controlled-release profiles with a tunable drug release amount in the second phase. The modified coaxial electrospinning is a useful tool to generate nanostructures with a tailored components and compositions in their different parts, and thus to realize the desired functional performances.

## 1. Introduction

Electrospinning, a simple and straightforward top-down process for generating nanofibers, has drawn increasing attentions due to easy implementation, capability of treating a variety of materials, convenience in obtaining composites of multiple components and a wide variety potential applications of the resultant nanofibers [1–6]. Coaxial electrospinning, in which a concentric spinneret can accommodate two liquids, is regarded as one of the most significant breakthroughs in this area [7–9]. The traditional concept about coaxial electrospinning is that “the shell solution is critical and the selected shell polymer-solvent system should be electrospinnable by itself to facilitate core–shell structure formation, whereas the core solution can either be electrospinnable or not” [10]. Through the application of this advanced process, a wide variety of possibilities for generating novel structural nanomaterials have been realized, such as encapsulating drugs or biological agents into polymer nanofibers, preparing nanofibers from materials that lack filament-forming properties, enclosing functional liquids within the fiber matrix [11–14].

However, a modified coaxial electrospinning, which is characterized by the usage of un-electrospinnable liquids as shell fluids, has been successfully developed for fabricating nanofibers [15,16]. Because there are many types of “unspinnable” liquids (solvents, solutions, suspensions and emulsions) that can be managed to act as shell fluids for conducting the modified coaxial process, thus this advanced process has greatly expanded the capability of the traditional coaxial electrospinning in generating novel nanostructures. Some examples include manipulating the size of self-assembled nanoparticles; preparing ultrafine fibers from concentrated polymer solutions thought to be un-spinnable previously; improving the quality of nanofibers systematically; and coating drug-loaded nanofibers with a blank release-retarded polymer layer for achieving zero-order drug release profiles [1,15–17].

In clinical applications, the desired drug release profiles should obey the biological rhythm for effective and safe drug delivery and convenient administration. For some active pharmaceutical ingredients (API) such as non-steroidal anti-inflammatory drugs (NSAIDs), as well as antihypertensive, antihistaminic and anti-allergic agents, an initial fast release of a fraction of the dose in the shortest time after administration is in favor of relieving the symptoms of the disease [17]. Meanwhile, a sustained release of the remaining dose over a defined period can optimize the therapy and avoid repeated administration for the patients’ convenience [18,19].

The dual release profiles often involve drug controlled release at two different rates or in two time periods. A typical dual release system can provide an immediate drug release followed by a constant release [20,21]. Because this type of controlled release profiles is very popular, a wide variety of technologies have been used to create drug delivery systems (SDS) furnishing dual release profiles. Over the past decades, different traditional pharmaceutical protocols such as tableting, casting, and spraying were investigated for preparing dual drug release DDS. Some of them include mixed films, multi-layered films, multiple tablets, multi-layered tablets, film-coated tablets, and eluting stents [18]. Meanwhile, advanced technologies (such as three-dimensional printing and nanotechniques [22,23]) are continuously investigated in the literature in order to produce novel materials or DDS for providing dual release, taking its advantage in a more accurate time-programmed administration of API and fulfilling the specific therapeutic needs of some diseases.

Electrospinning can achieve this objective through strategies such as preparing multi-layered nanofiber mats using a sequential single fluid process or producing fibers containing drug-loaded nanoparticles through the electrospinning of suspensions [23]. Core-shell nanofibers generated using coaxial electrospinning/triaxial electrospinning have also been reported to offer dual-stage drug release/dual-drug controlled release. In the former case, the shell delivers immediate release, followed by sustained release from the core [17,24]. Generally, both the core and shell fluids used for coaxial electrospinning were electrospinnable, limiting the possibilities for developing novel nanostructures from systems with poor or no electrospinnability.

Based on the above-mentioned knowledge, herein for the first time, we report a new type of drug-loaded core-shell nanofibers capable of providing dual controlled release with tunable dose in the second phase. Using poly(vinyl pyrrolidone) (PVP) and ethyl cellulose (EC) as the shell and core polymer matrices, and acetaminophen (APAP, a common NSAID) as the model API, a modified coaxial electrospinning with a Teflon-coated concentric spinneret was conducted for the preparation of core-shell drug-loaded nanofibers, in which the shell fluid was un-spinnable.

## 2. Results and Discussion

### 2.1. The Teflon-Coated Concentric Spinneret and the Modified Coaxial Electrospinning

**Figure 1.**
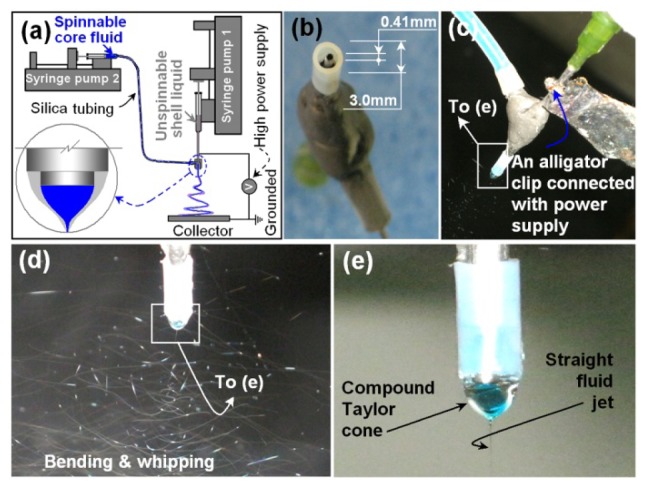
Implementation of the modified coaxial electrospinning processes using a concentric Teflon spinneret. (**a**) A diagram of the modified coaxial process; (**b**) Digital images of the homemade concentric Teflon-coated spinneret; (**c**) Connection of the power supply with spinneret; (**d** and **e**) Observations of the coaxial electrospinning processes: a typical fluid jet traveling process and a typical compound Taylor cone under a voltage of 15 kV and at a fiber collected distance of 20 cm for fabricating nanofibers F4.

A schematic diagram of the modified coaxial electrospinning process is shown in Figure 1a. The digital images at Figure 1b show the sizes of the concentric Teflon-coated spinneret used. The spinneret had an outer Teflon tubing with (Ø 3.0 mm × 0.5 mm) and an inner stainless-steel tubing (Ø 0.73 mm × 0.16 mm). The inner tubing projected out of the outer tubing by 0.5 mm. During the coaxial electrospinning process, the Teflon coating can facilitate the electrospinning on several aspects: (1) As an antistatic polymer, Teflon can effectively retard the loss of electrical energy from the working fluid to the environment; (2) The small interfacial tension between the inner wall of the Teflon tube and the working fluid exerts less negative influence of back-drawing on the formation of the compound Taylor cone and emission of the straight fluid jets; and (3) The outer surface of the Teflon tube makes it less impossibility for the possible “semi-solid” substance formed during electrospinning to cling on the spinneret to clog it [25].

Two syringe pumps were separately used to drive the shell and core fluids, and an alligator clip was used to connect the inner stainless-steel capillary of the spinneret to the high-voltage power supply (Figure 1c). Under the selected conditions of electrospinning for preparing core-shell nanofibers, the coaxial electrospinning processes were conducted smoothly and continuously, a typical fluid jet trajectory and a typical compound Taylor cone of the core-shell fluids are shown in Figure 1d,e. In the coaxial electrospinning system, a single fluid electrospinning could be carried out simply through adjusting one of the core or shell fluid flow rate to zero. The core drug-EC co-dissolving fluid had fine electrospinnability although clogging of the spinneret occurred now and then and human intervention was needed to remove the semi-solid substance on the spinneret. The shell solution, with a DMAc content of 20% (*v*/*v*), could not be electrospun into nanofibers or electrosprayed into microspheres.

In this study, four types of drug-loaded solid nanofibers were successfully produced and some key parameters are listed in Table 1.

### 2.2. Morphology and Structure of Nanofibers

**Figure 2.**
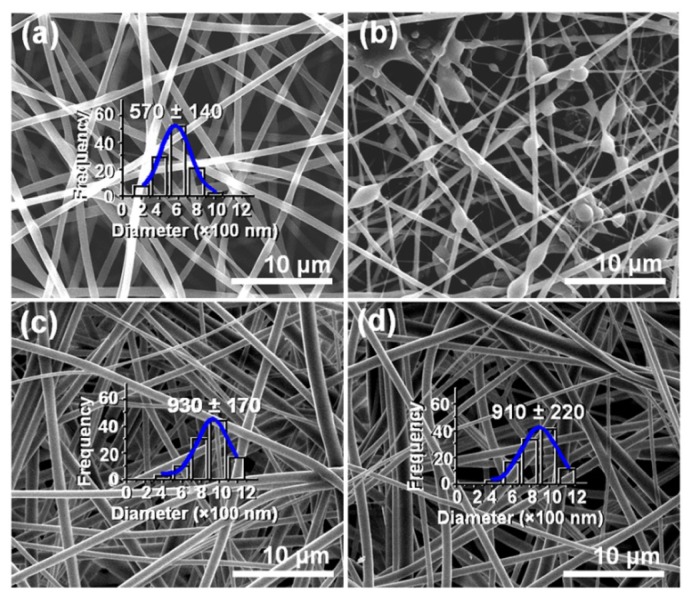
FESEM images of the electrospun nanofibers and their diameter distributions: (**a**) F1; (**b**) F5; (**c**) F3; (**d**) F4.

The morphologies of the four electrospun products are shown in Figure 2. Nanofiber F1 from the core solution using a single fluid electrospinning exhibited linear morphologies without spindles or beads-on-a-string morphologies and had an average diameter of 570 ± 140 nm (Figure 2a). The shell fluids resulted in no solid products because (1) the PVP K25 has a relatively small molecular weight; and (2) DMAc, an aprotic solvent, has a high boiling point of 166 °C with a low conductivity (0.31 mS·cm^−1^) [16]. The former could not provide enough polymer chain entanglements to subdue Rayleigh instability and the latter resulted in that the contained solvent could not evaporate duly and effectively. Even a big shell-to-core fluid flow rate ratio of 0.6 (0.3/0.5 mL/h) would deteriorate the quality of resultant nanofibers to degrade into beads or spindles-on-a-string morphologies, as indicated in Figure 2b. However, a certain content of DMAc and the usage of Teflon as nozzle material could ensure a smooth and continuous coaxial electrospinning process through retarding the occurrence of clogging. Provided the selection of shell-to-core fluid flow rate ratio was suitable, higher quality core-shell nanofibers were able to generate, as demonstrated by the productions of nanofibers F3 and F4 (Figure 2c,d), which had an average diameter of 930 ± 170 and 910 ± 220 nm, respectively.

**Figure 3.**
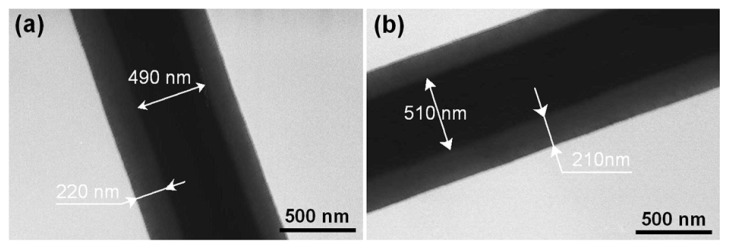
TEM images of the core-shell nanofibers: (**a**) F3 and (**b**) F4.

The nanofibers F3 and F4 had obvious core-shell structures, with an estimated core diameter and sheath thickness of 490 and 220 nm for F3 (Figure 3a) and a value of 510 and 210 nm for F4 (Figure 3b). Similar to the FESEM observations, no nanoparticles were discerned in the shell and core parts. This finding suggests that these nanofibers have a homogeneous structure. Although the shell fluid was un-spinnable, the fast drying electrospinning process similarly propagated the physical state of the components in the liquid solutions into the solid nanofibers and duplicated the concentric structure of the spinneret on a macroscale to products on a nanoscale. These two types of nanofibers had similar diameters and shell/core sizes, reflecting that the increase of drug concentration in the core fluid resulted in little difference.

### 2.3. Physical Status and Compatibility of Components

**Figure 4.**
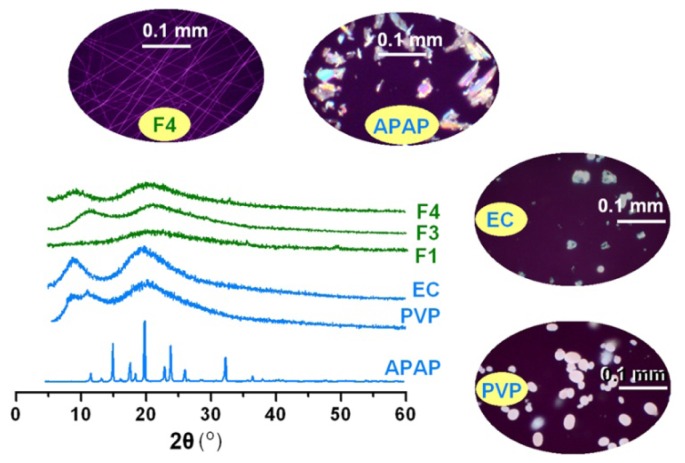
Physical status characterization: XRD patterns of the raw materials (ethyl cellulose (EC), poly(vinyl pyrrolidone) (PVP), and acetaminophen (APAP)) and the drug-loaded nanofibers: F1 (prepared by single fluid electrospinning), F3 and F4 (prepared by coaxial electrospinning).

XRD analyses were performed to determine the physical status of APAP in the core-shell nanofibers. As shown in Figure 4, the presence of many distinct peaks in the XRD patterns indicated that APAP was present as crystalline materials with characteristic diffraction peaks, as also demonstrated by the colorful images of their crude particles under polarized light. PVP and EC diffraction exhibited a diffused background pattern with two diffraction halos indicating that the polymers were amorphous. The patterns of nanofibers F1, F3 and F4 showed no characteristic reflections of the crude components and instead comprised diffuse haloes. Unlike the observations on APAP crystal particles, PVP, EC and the nanofibers F4 showed no any bright colors, suggesting they were amorphous. These results suggested that APAP were no longer present as a crystalline material but had been converted into an amorphous state both in the inner core with EC and in the outer shell with PVP. Electrospinning is an inherently appropriate method for preparing solid dispersion of poorly water soluble drugs for enhancing their solubility [9]. The fast drying electrospraying process can randomly “freeze” the drug molecules in the solid polymer matrix into a state comparable to a liquid form. This can prevent the solid phase separation, e.g., recrystallization of either drug or matrix, during solvent removal.

**Figure 5.**
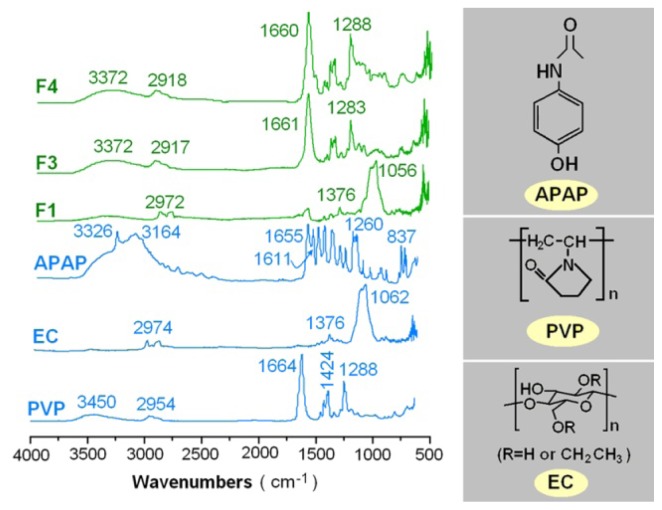
Compatibility investigation: ATR-FTIR spectra of the components (APAP, EC and PVP) and their electrospun nanofibers F1 (prepared by single fluid electrospinning) and F3, F4 (prepared by coaxial electrospinning) and the molecular structures of PAP, EC and PVP.

Compatibility between components is essential for producing high-quality and stable composite nanofibers. Second-order interactions such as hydrogen bonding, electrostatic interactions, and hydrophobic interactions can often improve compatibility [26]. The molecular structures of the three components are shown in Figure 5. In the FTIR spectrum of pure APAP, the peak at 3326 cm^−1^ is assigned to the N–H stretching vibration, and the peaks around 3164 cm^−1^ might be due to the OH stretching vibration plus other combination bands. The peaks at 1655 and 1260 cm^−1^ are assigned to the C=O stretching vibration of amide I band and/or C–N stretching of amides II and III vibrational bands, respectively. The peaks at 1611 cm^−1^ is due to the C=C bond stretching of aromatic benzene ring [27,28]. The spectrum of PVP K25 showed important bands at 2954 cm^−1^ (C–H stretch) and 1664 cm^−1^ (C=O).

The sharp peak of 3326 cm^−1^ and peaks around 3164 cm^−1^ in the pure APAP spectrum have changed to broad but weak bands in the nanofibers F1, F3 and F4. The characteristic peaks of APAP corresponding to the C=C stretching of the aromatic benzene ring attenuated, shifted, or even disappeared in the nanofibers’ spectra. In the fingerprint regions, almost all of the peaks of APAP were shifted, decreased in intensity, or even disappeared from the spectra of the three nanofibers. All these were attributed to the hydrogen bonding between the hydroxyl group of APAP and the carboxyl group of PVP in the outer shell of the nanofibers, or the hydrogen bonding between the hydroxyl group of EC and the carboxyl group of APAP in the inner core of the nanofibers. These favorite second-order interactions were not only useful for the formation of amorphous drug-loaded nanocomposites during the electrospinning processes, but also desired for preservations of these nanoproducts for aftertreatment and further development of DDS.

### 2.4. Dual Release Profiles with Tunable Dose in the Second Phase

APAP has a UV absorbance peak at λ_max_ = 257 nm. Thus, the amount of APAP released from the fibers was determined by UV spectroscopy using a predetermined calibration curve *C* = 13.37*A* – 0.2115 (*R* = 0.9996) where *C* is the concentration of APAP (μg/mL) and *A* is the solution absorbance at 257 nm (linear range: 2 to 20 μg/mL).

**Figure 6.**
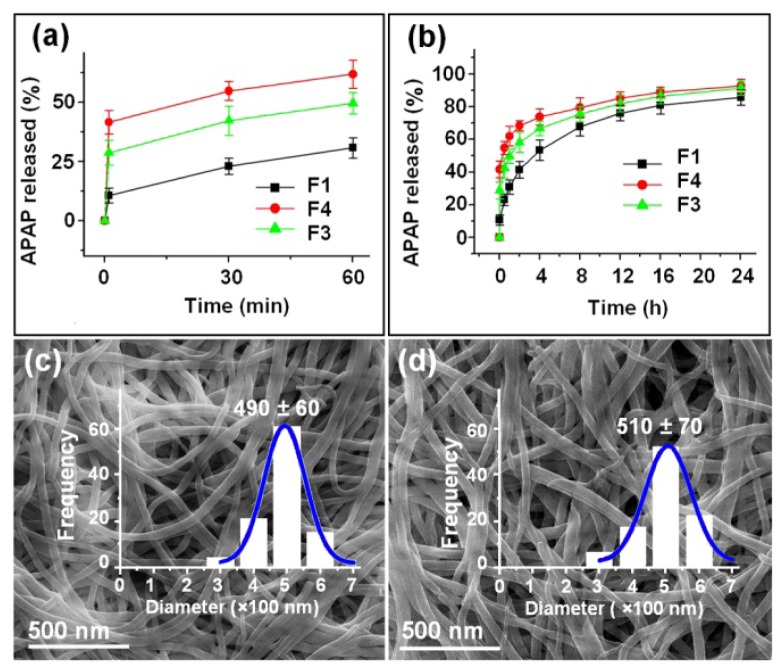
*In vitro* dissolution tests: (**a** and **b**) *In vitro* drug release profiles of the APAP-loaded nanofibers F1 (prepared by single fluid electrospinning), F3, and F4 (prepared by coaxial electrospinning) at the first 60 min and the full range, respectively; (**c** and **d**) FESEM images and size distribution of the core part of nanofibers F3 and F4 after the removal of the shell part in the first phase.

The actual content of APAP in all the nanofibers was equivalent to the theoretical calculation value, suggesting little drug loss during the electrospinning process. The *in vitro* drug release profiles of the three types of nanofibers are shown in Figure 6 and Table 2. As expected, the nanofibers of F3 and F4 could provide typical dual drug release profiles with immediate release percentages of 41.6% and 28.7%, respectively, after they were placed in the dissolution media for 1 min. The theoretical contents of APAP in the shell parts of nanofibers F3 and F4 were 33.3% and 20.0%, respectively, reflecting that not only all the drugs in the shell part were freed into the dissolution media but also a percentage of 8.3% and 8.7% of the drug contained in the core parts were released. This should be a result of the amorphous status of APAP, its uniform distributions in the core parts and a large outer surface of the core parts of the core-shell nanofibers. In contrast, the monolithic drug-EC nanofibers F1 released 11.3% of the contained drug in the first minute and a percentage of 30.8% in the first hour, suggesting an obvious initial burst release effect, which was out of control.

Later nanofibers F3 and F4 released 51.1% and 62.7% of the remaining APAP sustainably for 24 h (Figure 6b and Table 2). The core parts of the nanofibers of F3 and F4 after removal of the shell APAP/PVP had a diameter distribution of 490 ± 60 nm (Figure 6c), and 510 ± 70 nm, respectively (Figure 6d), almost the same with the TEM observations. The surfaces of the nanofibers remained smooth without discerned nanoparticles, similarly suggesting that APAP in the shell part was freed into the dissolution medium synchronously with the matrix PVP through a polymer-controlled erosion mechanism.

Thus, the dual release profiles could be achieved through a core-shell structure, wherein the shell hydrophilic polymer provides a pulsatile release initially and the core matrix furnishes the later sustained release. Previous reports have also demonstrated the usefulness of core-shell structures in providing this type of controlled release profiles [17,29]. However, this work shows more superior to them in that: (1) The drug release in the second phase can be tunable simply through the usage of a higher concentration drug-loaded core solution. Here, with a same total drug amount of 20 mg, the nanofibers F4 released 62.7% of the contained drug, more than the value of 51.1% by nanofibers F3. Previous study demonstrated that the amount of drug released could be controlled by adjusting the shell flow rate [29], however, this change of the electrospinning parameter would complicate the processes due to the un-spinnable shell solutions; (2) A modified coaxial electrospinning process was developed, in which un-spinnable solutions can be taken as shell fluid, expanding the capability of the traditional coaxial processes, in which only electrospinnable liquids can be exploited as shell fluids and very limited polymer systems have electrospinnability; (3) The reasonable selection of solvent, *i.e.* a mixed solvent with the addition of DMAc would effectively prevent the clogging of spinneret due to its high boiling point; and (4) The usage of PVP k25 would promote the fast exhaustion of the shell drug than its analogues such as PVPK30, PVP K60 and PVPK90. The amorphous APAP dissolved in the dissolution medium through the erosion of polymer matrix PVP. Often the polymer dissolution involve two sequential processes, *i.e.*, absorbing solvent to form a swollen gel, and then followed by chain disentangles and disperses into the solution of the swollen polymer. Thus, a higher molecular weight meaning a longer time period of dissolutions and the drug dissolution processes. Here, the PVP K25, although could not be electrospun into nanofibers or electrosprayed into microparticles, but could be solidified as shell parts based on the electrospinnable core fluids and it could enhance the fast dissolution of APAP. In a word, for the first time, the drug concentrations in the core fluids were exploited as a useful tool to tailor the dose of drug released in the second phases.

## 3. Experimental Section

### 3.1. Materials

APAP (purity > 99%) was obtained from the 4th Pharmaceutical Factory of Weifang (Weifang, Shandong, China). PVP K25 (*M*_w_ = 30, 000) was purchased from Shanghai Yunhong Pharmaceutical Aids and Technology Co., Ltd. (Shanghai, China). EC (6 to 9 mPa·s) was obtained from Aladdin Chemistry Co., Ltd. (Shanghai, China). Methylene blue, *N*,*N*-dimethylacetamide (DMAc), and anhydrous ethanol were purchased from Sinopharm Chemical Reagent Co. Ltd. (Shanghai, China). All other chemicals used were analytical grade. Water was immediately double distilled before use.

### 3.2. Modified Coaxial Electrospinning

The shell fluid consists of 15% (*w*/*v*) PVP K25 and a fixed APAP concentration of 5% (*w*/*v*) in a mixture of ethanol and DMAc (8:2, *v:v*); The core fluid consists of 24% (*w*/*v*) EC and a varied concentration of APAP in ethanol, as described in Table 1. To observe the electrospinning process, 5 ppm methylene blue was added to the core solution. A homemade Teflon-coated concentric spinneret was used to conduct the coaxial electrospinning processes. Two syringe pumps (KDS100 and KDS200, Cole-Parmer, Vernon Hills, IL, USA) and a high-voltage power supply (ZGF 60 kV, 2 mA^−1^, Shanghai Sute Corp., Shanghai, China) was used for electrospinning, which was performed under ambient conditions (23 ± 4 °C; 61% ± 4% relative humidity). The modified coaxial electrospinning processes were recorded using a digital video recorder (PowerShot A490, Canon, Tokyo, Japan). After optimization, the applied voltage was fixed at 15 kV and the fibers were collected on an aluminum foil 20 cm from the spinneret. All other parameters are listed in Table 1.

### 3.3. Characterization

#### 3.3.1. Morphology

The morphology of the nanofibers was examined using an S-4800 field-emission scanning electron microscope (Hitachi, Tokyo, Japan). Prior to examination, the samples were subject to platinum sputter-coating in nitrogen atmosphere for electrical conductivity. The average fiber diameter was determined by measuring their sizes in FESEM images at more than 100 different places using NIH Image J software (National Institutes of Health, Bethesda, MD, USA). TEM images of the samples were recorded on a JEM 2100F field-emission instrument (JEOL, Tokyo, Japan). TEM images of the core-shell nanofibers were obtained by fixing a lacey carbon-coated copper grid on the collector. The topographies of the raw particles of APAP, EC and PVP, and also the nanofibers F4 was observed under cross-polarized light using an XP-700 polarized optical microscope (Shanghai Changfang Optical Instrument Co., Ltd., Shanghai, China).

#### 3.3.2. Physical Status and Compatibility

XRD patterns were obtained over the 2θ range of 5° to 60° on a D/Max-BR diffractometer (RigaKu, Tokyo, Japan) with Cu Kα radiation at 40 mV and 30 mA. FTIR analysis was conducted on a Nicolet-Nexus 670 FTIR spectrometer (Nicolet Instrument Corporation, Madison, WI, USA) from 500 to 4000 cm^−1^ at a resolution of 2 cm^−1^.

#### 3.3.3. *In Vitro* Dissolution Tests

*In vitro* dissolution tests were carried out according to the Chinese Pharmacopoeia, 2005 ed. Method II, a paddle method, was performed using a RCZ-8A dissolution apparatus (Tianjin University Radio Factory, Tianjin, China). An equal amount APAP of 20 mg (*i.e.*, 180 mg F1, 147 mg F3 and 96 mg F4) were placed in 600 mL of physiological saline (PS, 0.9 wt %) at 37 ± 1 °C. The instrument was set to stir at 50 rpm, providing sink conditions with *C* < 0.2*C*_s_. At predetermined time points, 5.0 mL aliquots were withdrawn from the dissolution medium and replaced with fresh medium to maintain a constant volume. After filtration through a 0.22 μm membrane (Millipore, Billerica, MA, USA) and appropriate dilution with PS, the samples were analyzed at λ_max_ = 257 nm using a UV/Vis spectrophotometer (UV-2102PC, Unico Instrument Co. Ltd., Shanghai, China). The accumulative APAP released was back-calculated from the data obtained against a predetermined calibration curve. The experiments were carried out six times, and the accumulative release amount reported as mean values was plotted as a function of time (*T*, min or h).

## 4. Conclusions

A modified coaxial electrospinning was carried out to prepare core-shell nanofibers that provided dual drug release profiles of APAP, and the drug contents in the core fluids were successfully exploited as a tool to tailor the dose of drug released in the second phase. A Teflon-coated concentric spinneret was used to carry out the modified coaxial electrospinning. Although the shell fluid had no electrospinnability, the addition of DMAc in the shell solvent system may facilitate the coaxial electrospinning process through prevent the clogging of spinneret, and the usage of PVP K25 with a relatively small molecules could promote the fast dissolution of APAP in the first immediate release phase. XRD results verified that APAP was present in an amorphous status in the outer shell with PVP and in the inner core with EC in the core-shell nanofibers. FTIR spectra demonstrated that the shell polymer and core EC matrix were compatible with APAP owing to hydrogen bonding. *In vitro* dissolution tests exhibited that the core-shell nanofibers could provide dual release profiles consisting of an immediate and a sustained release. The amount of drug released in the second phase was tunable. The present study showed a simple and useful approach for the systematic design and fabrication of novel biomaterials with structural characteristics for providing complicated and programmed drug release profiles using the modified coaxial electrospinning.

## Figures and Tables

**Table 1.** Parameters used for electrospinning and details of the nanofiber products.

No.	Process	Drug con. (*w*/*v*)		Flow rate (mL/h)		Morphology c	Total drug content d	Diameter (nm)
	
		Shell a	Core b	Shell	Core			
F1	Single	-	3%	-	1.0	Linear	11.11%	570 ± 140
F2		5%	-	1.0	-	-	-	-

F3	Coaxial	5%	3%	0.3	1.0	Linear	13.64%	930 ± 170
F4		5%	6%	0.3	1.0	Linear	20.83%	910 ± 220
F5		5%	6%	0.3	0.5	Mixed	-	-

a Shell fluid consists of 15% (*w*/*v*) PVP K25 and a fixed APAP concentration of 5% (*w*/*v*) in a mixture of ethanol and DMAc (8:2, *v:v*);

b Core fluid consists of 24% (*w*/*v*) EC and a varied concentration of APAP in ethanol;

c “Linear” morphology refers to nanofibers with few beads or spindles and “Mixed” morphology to nanofibers exhibiting beads-on-a-string or spindles-on-a-string features;

d The theoretical value (*w*/*w*) in the solid nanofibers calculated according to the shell and core fluid flow rates and the drug content in the shell and core solutions.

**Table 2.** Comparison of the release parameters of the nanofibers (*n* = 6).

Nanofiber No.	Release in the 1st phase (1 min)		Release after 24 h b	Release in the 2nd phase	
	
	Theoretical a	Experimental b		Theoretical a	Experimental b
F1	-	10.7% (2.1)	85.4% (17.1)	-	74.7% (14.9)
F3	33.3% (6.7)	41.6% (8.3)	92.7% (18.5)	66.7% (13.3)	51.1% (10.2)
F4	20.0% (4.0)	28.7% (5.7)	91.4% (18.3)	80.0% (16.0)	62.7% (12.5)

a Calculated according to the equation: *P* = (*F*_s_ × *C*_s_)/[(*F*_s_ × *C*_s_) + (*F*_c_ × *C*_c_)]. *F*_s_, *F*_c_, *C*_s_ and *C*_c_ represent the flow rates and drug contents of the sheath and core fluids, respectively;

b Expressed as mean values and the numbers in the brackets represents the absolute drug release amount in mg.
